# Two or Four Bristles: Functional Evolution of an Enhancer of *scute* in Drosophilidae

**DOI:** 10.1371/journal.pbio.0040386

**Published:** 2006-11-14

**Authors:** Sylvain Marcellini, Pat Simpson

**Affiliations:** Department of Zoology, University of Cambridge, Cambridge, United Kingdom; Duke University, United States of America

## Abstract

Changes in *cis-*regulatory sequences are proposed to underlie much of morphological evolution. Yet, little is known about how such modifications translate into phenotypic differences. To address this problem, we focus on the dorsocentral bristles of Drosophilidae. In *Drosophila melanogaster,* development of these bristles depends on a *cis-*regulatory element, the dorsocentral enhancer, to activate *scute* in a cluster of cells from which two bristles on the posterior scutum arise. A few species however, such as *D. quadrilineata,* bear anterior dorsocentral bristles as well as posterior ones, a derived feature. This correlates with an anterior expansion of the *scute* expression domain. Here, we show that the D. quadrilineata enhancer has evolved, and is now active in more anterior regions. When used to rescue *scute* expression in transgenic *D. melanogaster,* the D. quadrilineata enhancer is able to induce anterior bristles. Importantly, these properties are not displayed by homologous enhancers from control species bearing only two posterior bristles. We also provide evidence that upstream regulation of the enhancer, by the GATA transcription factor Pannier, has been evolutionarily conserved. This work illustrates how, in the context of a conserved *trans-*regulatory landscape, evolutionary tinkering of pre-existing enhancers can modify gene expression patterns and contribute to morphological diversification.

## Abstract 2

Evolutionary change in function of the dorsocentral enhancer (DCE) of scute has resulted in altered bristle formation between two species of Drosophila.

## Introduction

Development is a complex process during which a plethora of regulatory mechanisms progressively unfold to ensure correct spatio-temporal expression of the genome. Morphological evolution occurs when mutations modifying these mechanisms produce a new phenotype, are tolerated, and fixed in a population [1,2]. Although phenotypic evolution could result from changes at many regulatory levels [3], there has been an ever-growing emphasis that modular *cis*-regulatory enhancers might be the major mutational targets [4–12]. A few studies correlate evolutionary changes in *cis*-regulatory regions to anatomical traits that differentiate species. Examples include linkage of the evolution of a *Hoxc8* enhancer with changes in vertebrate axial identity, of a *lin-48* enhancer with modifications of the nematode excretory duct, and of *yellow* enhancers with diversification of pigment patterns in Drosophilidae [13–17]. Functional tests of other cases would help decipher the complex relationship existing between evolution of *cis-*regulatory sequences and morphological evolution.

Within dipteran flies, bristle patterns are variable, but often stereotyped and species specific [18]. Indeed within the Schizophora, a monophyletic group of the Diptera, the large bristles, macrochaetes, can be homologised. The genetic basis of bristle development in Drosophila melanogaster has been intensively investigated over several decades [19,20]. The positions of bristles on the thorax depend on the precise spatial expression of the *achaete-scute (ac-sc)* genes, mediated by numerous independently acting enhancers [21,22]. Bristle patterns therefore offer an ideal paradigm to study evolutionary changes in gene regulation. Within the Schizophora, different patterns correlate with changes in *sc* expression [23–26]. Such changes could result from alterations in *trans-*acting factors or to *cis-*regulatory changes at the *sc* locus itself. The expression domains of the *trans-*acting factors are unchanged between Calliphora vicina and D. melanogaster suggesting conservation of a *trans-*regulatory gene network throughout the 100 million years (Myr) of evolution separating these two species [27]. This prompts investigation of *cis-*regulatory sequences.

The proneural *ac-sc* genes are expressed in small clusters of cells on the notum, proneural clusters, at the sites of formation of bristle precursors [28,29]. Expression is mediated by a number of enhancer modules of which one, the dorsocentral enhancer (DCE), has been characterized in some detail. It interacts with the GATA transcription factor Pannier (Pnr). Pnr binds this element and loss of function of *pannier (pnr)* abolishes *sc* expression at the dorsocentral (DC) site [21,30–32]. Furthermore, mutation of GATA sequences shown to bind Pnr causes a loss of enhancer activity when assayed in reporter gene constructs [30]. Here we have analyzed the activity of this enhancer from other species of Drosophilidae with variable numbers of dorsocentral bristles to examine its possible evolution. First, we show that, despite significant sequence turnover, its function has been retained between species with a divergence time of up to 60 Myr, that, like *D. melanogaster,* bear two DC bristles. Second, we demonstrate functional evolution of the enhancer in a species with four to five DC bristles.

## Results

### A Secondary Gain of Anterior DC Bristles in *D. quadrilineata*

**Figure 1:**
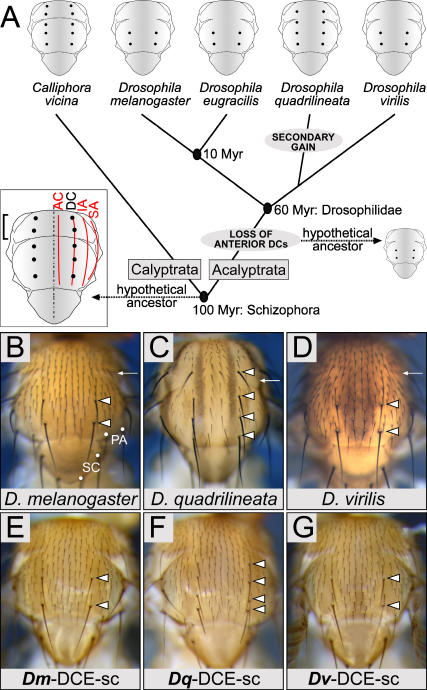
The Number of DC Bristles Correlates with DCE Activity (A) Phylogenetic relationships of the species used in this study. Available estimates for the divergence times are indicated. The last common ancestor of the Schizophora possessed four longitudinal rows of macrochaetes per heminotum (red lines). The dotted line indicates the midline. For clarity, only the DC bristles are shown (black dots). Note the presence of anterior bristles within the presutural region (bracket), a trait inherited by many extant Calyptrata (e.g., *Calliphora vicina*). *D. virilis, D. melanogaster,* and many other species of Acalyptrata exhibit only posterior DC bristles, due to the extensive loss of presutural macrochaetes in this group. In the lineage leading to *D. quadrilineata,* two additional anterior DC bristles have emerged through secondary gain. AC, acrostichal; DC, dorsocentral; IA, intraalar; SA, supraalar. (B–D) Dorsal views of adult thoraces from D. melanogaster (B), D. quadrilineata (C), and *D. virilis* (D). White arrows indicate the transverse suture. In (B), white dots indicate the scutellar (SC) and postalar (PA) bristles. (E–G) Dorsal views of adult thoraces from D. melanogaster flies carrying one copy of the *Dm*-DCE-sc, *Dq*-DCE-sc, and *Dv*-DCE-sc transgenes, respectively. White arrowheads indicate DC bristles. Note the presence of anterior DC bristles in (C) and (F).

The last common ancestor of the Schizophora is thought to have possessed four longitudinal rows of bristles extending from anterior to posterior on the scutum [18,33,34]. The Schizophora comprises the calyptrate and acalyptrate lineages, and many species of Calyptrata retain four complete bristle rows (Figure 1A). The Acalyptrata display reduced, derived patterns due to partial or complete loss of rows [18,33]. Bristle loss is most frequent on the anterior notum [35]. Absence of the anterior bristles of the DC row, particularly those situated in the prescutum anterior to the transverse suture is an apomorphic character found in many Acalyptrata [18,35]. Indeed the presence of only two, posteriorly situated DC bristles is a plesiomorphic feature of the family Drosophilidae [36]. Thus many extant and extinct *Drosophila* species display two posterior DC bristles at stereotyped locations (Figure 1) [35–37]. This is the case for D. virilis and *D. melanogaster,* for example, separated by 60 Myr of independent evolution (Figure 1A, 1B, and 1D). A few *Drosophila* species, like *D. quadrilineata,* display anterior DC bristles on the scutum as well as on the prescutum, thereby mimicking the ancestral situation of the Schizophora (Figure 1A and 1C). These are thought to have arisen by secondary gain. D. quadrilineata belongs to the *immigrans* subgroup [38,39], implying that it is more closely related to D. virilis than it is to D. melanogaster [40].

### The DCE from D. quadrilineata Allows Development of Four DC Bristles in D. melanogaster

To examine whether the mutation(s) responsible for the re-emergence of anterior DC bristles reside in the sequence of the DCE of D. quadrilineata (*Dq-*DCE), we constructed *sc* minigenes. It has previously been demonstrated that, when used to drive the expression of a *sc* minigene in an *ac-sc* null mutant background, the DCE of D. melanogaster (*Dm-*DCE) is sufficient to rescue the formation of the two posterior DC bristles [41]. Following a similar strategy, we compared orthologous DCEs from species with two DC bristles, D. melanogaster and *D. virilis* (*Dv-*DCE), or four DC bristles, D. quadrilineata. We subcloned the enhancers upstream of a *sc* minigene, and generated four independent insertion lines for each transgene in D. melanogaster hosts. When assayed in a wild-type background, all lines bearing *Dm-*DCE-*sc* and *Dv-*DCE-*sc* display an unchanged pattern of two posterior DC bristles (Figure 1E and 1G). Remarkably, one third of the flies bearing *Dq-*DCE-*sc* (four independent lines were examined) exhibit a row of four DC bristles that include bristles in a more anterior location (Figure 1F), thereby mimicking the phenotype of D. quadrilineata itself (Figure 1C).

**Figure 2:**
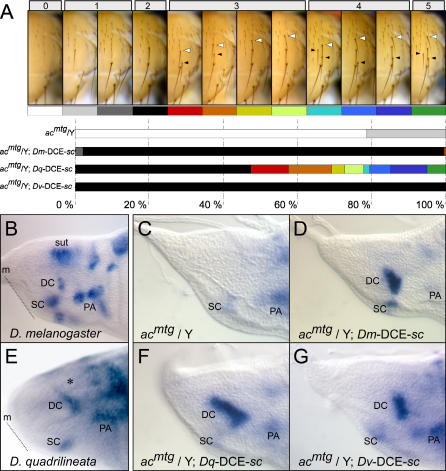
Rescuing Activity of *scute* Minigenes Minigenes comprising the enhancers of *D. melanogaster, D. quadrilineata,* and D. virilis and *sc (Dm-*DCE-*sc, Dv-*DCE-*s,c* and *Dq-*DCE-*sc)* were assayed in *ac^mtg^* mutants. (A) Pictures of typical adult hemithoraces characterised by the total number of bristles (indicated at the top), the position of the anterior-most bristle (white arrowheads), and of the smaller intermediate bristles (black arrowheads). Each hemithorax category is given a colour code below. The associated histograms show the percentage of hemithoraces falling in the above categories for the genotypes examined. This summarises the results from four independent insertion lines of each transgene, with at least 100 hemithoraces scored for each line. (B–G) In situ hybridisation for *sc* performed on third instar larval wing discs of the genotypes indicated. In *D. quadrilineata,* we observed expression of *sc* at the site of origin of the DC, scutellar (SC), and postalar (PSA) bristles, but noted its absence at the presutural position (see asterisk [*] in [E]). D. quadrilineata flies lack the presutural bristle (arrows in Figure 1B–1D). Expression corresponding to the presutural bristles of D. melanogaster is labelled “sut” in (B). The dotted lines in (B) and (E) indicate the midline (m). Anterior is up, posterior down, dorsal to the left, and lateral to the right.

To rigorously compare the ability of homologous DCEs to rescue DC bristle formation in D. melanogaster hosts, we took advantage of *Df(1)91B,* a 45-kilobase (kb) deletion that removes *ac* and the DCE, but leaves *sc* and most other regulatory elements intact [42]. This viable recessive mutant, hereafter referred to as *ac^mind the gap^ (ac^mtg^)*, exhibits a dramatic decrease in DC bristles: 80% of the scored hemithoraces are devoid of them entirely (Figure 2A). A single copy of the *Dm-*DCE-*sc* or *Dv-*DCE-*sc* minigenes rescues the two posterior DC bristles characteristic of both D. melanogaster and D. virilis in nearly all flies (Figure 2A). A single copy of *Dq-*DCE-*sc* also rescues DC bristles: in a little less than half the cases, there are two bristles, but in the others, there are three to five bristles (Figures 2A and S1). Note that some D. quadrilineata flies bear five DC bristles. Despite the expected variability between different insertion lines, the weakest *Dq*-DCE-*sc* strain still rescues more bristles than the *Dm*-DCE-*sc* or *Dv*-DCE-*sc* controls (Figure S1). Interestingly, the bristles are usually aligned, and extra bristles are mainly situated at more anterior locations on the scutum, sometimes at the level of the transverse suture (white arrowheads in Figure 2A). Bristles anterior to the transverse suture were not observed. Hence, the *Dq*-DCE-*sc* minigene is sufficient to confer on *D. melanogaster,* at least partially, a phenotype characteristic of D. quadrilineata.

### The *Dq-*DCE Drives Expression in a Longitudinal Stripe Extending Anteriorly

To understand how homologous DCEs promote the emergence of different bristle patterns, we compared the expression of the proneural gene *sc* by in situ hybridisation in third larval instar wing discs of wild-type flies and transgenic *ac^mtg^* mutants (Figure 2B–2G). In *D. melanogaster,* the proneural cluster that gives rise to the DC bristles is oval in shape with its long axis orientated roughly parallel to the antero-posterior axis (Figure 2B). By contrast, in *D. quadrilineata, sc* is expressed in the region corresponding to the site of origin of the DC bristles in a streak of cells that is elongated anteriorly (Figure 2E). This elongated proneural cluster does not extend in a straight line, but makes a sharp turn to become parallel to the midline (Figure 2E). As expected, *sc* expression is undetectable in the DC cluster of hemizygous *ac^mtg^* wing discs (Figure 2C). It is expressed in the other proneural clusters, such as the SC and PA, although at weaker levels than the wild type (Figure 2B and 2C). One copy of the *Dm-DCE-sc* or *Dv-DCE-sc* minigene restores the expression of *sc* in an oval-shaped cluster in *ac^mtg^* mutants (Figure 2D and 2G). The cluster rescued by the *Dq-*DCE-*sc* transgene, however, displays *sc* expression in an elongated streak, extending anteriorly and following the midline of the disc. This is similar to endogenous *sc* expression in D. quadrilineata wing discs (compare Figure 2E with 2F). Our results reveal that functional changes within the *Dq*-DCE are sufficient to confer upon *D. melanogaster,* a bristle pattern typical of *D. quadrilineata* resulting from a proneural cluster of elongated shape. Importantly, these properties are unique to the *Dq*-DCE because they are not displayed by the controls *Dm*-DCE and *Dv*-DCE.

### Pairwise Comparison of the Activity of Orthologous DCEs

Although the in situ hybridisation for *sc* revealed functional divergence between homologous DCEs (Figure 2), only double stainings performed at cellular resolution can provide an accurate comparison of enhancer activity. It has previously been shown that the *Dm-*DCE drives the expression of a cytoplasmic form of ß-Gal in cells of the DC proneural cluster [30]. We have used this *lacZ* reporter line as an internal reference, and compared it to other DCEs driving the expression of a nuclear form of GFP. In addition to *Dq-*DCE and *Dv-*DCE, we have included the DCE from D. eugracilis (*De*-DCE), whose phylogenetic position is closer to D. melanogaster (Figure 1) [40,43].

**Figure 3:**
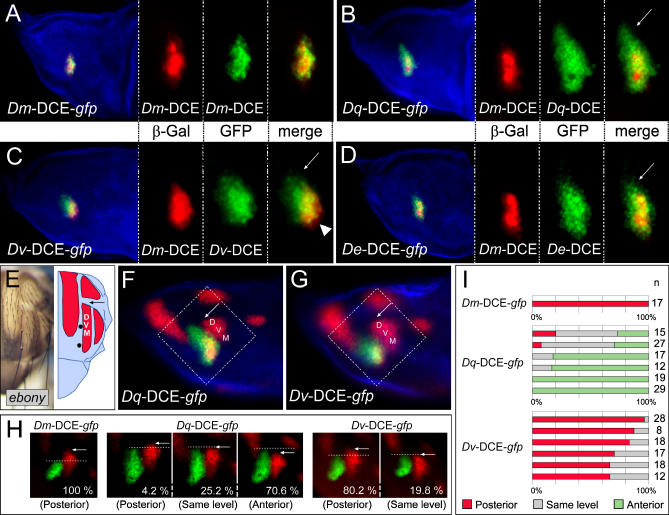
Detailed Comparison of the Activity of Orthologous DCEs (A–D) *D. melanogaster* wing discs expressing a cytoplasmic form of ß-Gal (red) under the control of the *Dm-*DCE, and a nuclear form of GFP (green) under the control of the DCE from D. melanogaster (A) *D. quadrilineata* (B), D. virilis (C), or D. eugracilis (D). A phalloidin counter-stain reveals actin in blue. In (B–D), the arrows point to anterior cells expressing GFP but not ß-Gal. In (C), a few posterior cells strongly express ß-Gal but weakly GFP (arrowhead). (E) Adult hemithorax of an *ebony* mutant, the muscle attachment sites appear as unpigmented cuticle. The schematic drawing shows the sites of muscle attachment (red) and the DC bristles (black dots). (F) and (G) ß-Gal antibody staining (red) revealing the expression of both *sr-lacZ* (enhancer trap line, nuclear signal) and of the *Dm*-DCE-*lacZ*. The expression of GFP driven by the DC enhancers of D. quadrilineata (F) or D. virilis (G) is shown in green. The regions indicated by the dashed boxes are shown in (H). In (E–G), the site of DVM 2–3 tendons is indicated (DVM). (H) Comparison of *sr* expression in the DVM (red) with the activity of the DCEs of *D. melanogaster, D. quadrilineata,* or D. virilis driving GFP (green). The dotted lines demarcate the anterior limit of activity of the DCEs. The regions shown are in a similar position to the dashed boxes in (F) and (G). In (E–H), the arrows point to the anterior limit of the DVM. The samples in (H) correspond to three categories depending on whether GFP signal was observed posterior to, at the same level as, or anterior to the anterior limit of DVM *sr* expression. The percentages of discs falling into each category are indicated. (I) The histograms detail the frequency of each category, for the DCE from D. melanogaster (one transgenic line), D. quadrilineata (six lines), or D. virilis (six lines). The numbers of discs examined per line are indicated (n).

We first verified that two independent transgenes of the *Dm-*DCE, driving expression of ß-Gal or GFP, are indeed active in precisely the same cells of the disc (Figure 3A). Expression driven by the orthologous DCEs overlaps in each case with the endogenous DC cluster, but the expression domains differ in detail. The pattern of GFP driven by the *Dq-*DCE extends significantly farther anteriorly than that of the *Dm-*DCE (arrow in Figure 3B). The *Dv-*DCE drives expression in a larger cluster of cells that overlaps only partly with the endogenous one: it is moderately displaced dorsally (arrowhead in Figure 3C) and anteriorly (arrow in Figure 3C). The *De-*DCE drives expression in a completely overlapping domain that is a little broader than the endogenous one and extends slightly anteriorly (arrow in Figure 3D).

Although the *Dv*-DCE drives the expression of a reporter gene in cells located more anteriorly than the endogenous DC cluster (Figure 3C), it is unable to induce the formation of anterior DC bristles (Figure 2A). To examine this in more detail, we compared the anterior limit of expression of the reporter genes with respect to an independent spatial reference. We focused on *stripe (sr)*, a gene expressed in four distinct domains in third larval instar wing discs from which tendon precursor cells are selected [44]. These domains are adjacent to the proneural clusters of *sc* expression [34]. The dorsoventral muscles 2 and 3 (DVM) tendons extend along the anterior–posterior axis, stopping just below the transverse suture (Figure 3E). The anterior limit of the expression domain of *sr* corresponding to the DVM precursors thus provides a sharp, reliable spatial landmark (arrows in Figure 3E–3H). We simultaneously compared *sr* expression with the activity of the *Dm-*DCE and *Dq-*DCE (Figure 3F) or of the *Dm-*DCE and *Dv-*DCE (Figure 3G). All three enhancers mediate expression in a cluster of cells abutting the dorsal aspect of the DVM *sr* expression domain (Figure 3F and 3G). Activity of the *Dv-*DCE spreads diffusely in a dorso-anterior direction (Figure 3G), whereas the *Dq-*DCE is active in an elongated cluster of cells that bends parallel to the DVM *sr* expression domain and to the dorsal midline of the disc (Figure 3F). The anterior limit of expression mediated by the *Dm-*DCE and the *Dv-*DCE in most of the cases analyzed, ends at a position posterior to the anterior limit of *sr* expression (Figure 3H and 3I). The *Dq-*DCE, on the other hand, reproducibly mediates GFP expression anterior to the anterior-most limit of *sr* expression (Figure 3H and 3I). Thus, the *Dq*-DCE drives expression more anteriorly than the *Dv*-DCE, and it is this feature that allows the *Dq*-DCE to induce the formation of anterior DC bristles up to the level of the suture.

### The Response of Orthologous Enhancers to Variations in *pnr* Activity

Unfortunately, to date, the mechanism responsible for restricting the activity of the DC enhancer in the anterior direction has not been discovered. However, the direct input of Pnr and U-shaped (Ush), essential for the correct activity of the *Dm*-DCE along the dorso-lateral axis, has been extensively analyzed. In order to shed light on the ancestry and the functional conservation of the regulation by Pnr and Ush, we compared the sequences of orthologous DCEs, as well as their relative activities in various mutant backgrounds.

**Figure 4:**
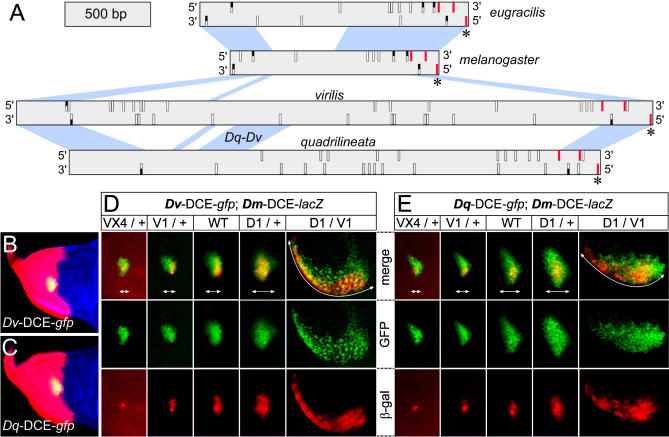
Divergent Enhancers Display a Similar Response to Pnr and Ush (A) Diagram representing the DCE sequences of *D. eugracilis, D. melanogaster, D. virilis,* and D. quadrilineata. The scale is shown at the top left. Only the blue regions connecting adjacent enhancers are alignable. A 300-bp region shared exclusively between D. quadrilineata and D. virilis is indicated *(Dq-Dv)*. Small vertical rectangles symbolise all the putative GATA sites found in the forward (top) or reverse (bottom) strand. They are white when they exist only in one species, red when found in all species, and half-black when shared by two or three species. The asterisks (*) mark a conserved Pnr binding site essential for normal activity of the D. melanogaster enhancer. (B) and (C) The D. virilis (B) and the D. quadrilineata (C) enhancers drive GFP expression (green) within the *pnr* expression domain, which is visualized by ß-Gal antibody staining of *pnr-Gal4/UAS-lacZ* wing discs (red). A phalloidin counter-stain reveals actin in blue. (D) and (E) Activity of the D. virilis (D) or D. quadrilineata (E) DCEs driving the expression of GFP (green) in the five genotypes indicated. In all cases, the activity of the D. melanogaster DCE driving expression of a cytoplasmic form of ß-Gal (red) was used as an internal reference. White double-headed arrows delimitate the dorso-lateral width of the clusters of cells expressing GFP. D1, *pnr^D1^;* V1, *pnr^V1^;* VX4, *pnr^VX4^;* WT, wild-type.

Sequence alignments reveal that the DCEs are greatly variable in size and have undergone considerable turnover. Only the extremities display significant levels of similarity between all species examined (shown in blue in Figure 4A). The central region is poorly conserved. The elements from *D. melanogaster* (1.5 kb) and D. eugracilis (2 kb) are more similar to each other than to the others, in accordance with their closer phylogenetic relationship (Figure 4A). The enhancers from *D. virilis* and D. quadrilineata share a relatively large size (4.1 and 3.3 kb, respectively) and a conserved stretch of about 300 nucleotides that is absent from the D. melanogaster and D. eugracilis sequences (labelled *Dq-Dv* in Figures 4A and S2). Putative binding sites for Pnr are present in all species (rectangles in Figure 4A). Mutation of a specific Pnr binding site severely reduces activity of the *Dm-*DCE [30]. This site is embedded within a stretch of 16 nucleotides perfectly conserved between the four species (asterisks in Figure 4A). Interestingly, two other neighbouring GATA sequences can be recognised as homologous between all species (red rectangles in Figure 4A). Conservation overall, however, is low, and the number, spacing, and orientation of the remaining putative Pnr binding sites are extremely variable (Figures 4A and S2).

In *D. melanogaster, pnr* is expressed in a broad medial domain, but activates *sc* in discrete proneural clusters [31]. Expression of *sc* mediated by the *Dm-*DCE is a direct consequence of Pnr binding [30]. DCE function is restricted dorsally through the repressor activity of Ush, which forms heterodimers with Pnr and prevents activation of *sc* [32,45]. We found that the activity of the *Dv-*DCE and the *Dq-*DCE in D. melanogaster is restricted to a lateral cluster of cells completely included within the expression domain of *pnr* (Figure 4B and 4C). This suggests that, despite significant sequence turnover, the divergent DCEs require Pnr and are efficiently repressed dorsally by Ush. We examined behaviour of the DCEs in the context of various mutant alleles of *pnr*. We used *pnr^VX4^,* a strong loss of function allele, *pnr^V1^,* a hypomorphic allele and *pnr^D1^,* a gain of function allele with a missense mutation that disrupts the interaction of Pnr with Ush [31,32,45]. Activity of the *Dm-*DCE was compared with that of the *Dv-*DCE (Figure 4D) and that of the *Dq-DCE* (Figure 4E). We observed that the enhancers react in a similar fashion to four different mutant backgrounds. The expression domains are reduced in loss of function genotypes and expanded in gain of function genotypes.

## Discussion

### A Conserved *trans-*Regulatory Landscape

To date, a single *trans-*regulator of the DCE, the GATA factor Pnr, has been identified in D. melanogaster [30,31]. We present evidence that the activity of Pnr is conserved and positively regulates the DC enhancers from distantly related Drosophilidae. When assayed in *D. melanogaster,* the *Dv-*DCE and *Dq-*DCE are active in groups of cells completely included within the expression domain of *Dm-pnr.* It is significant that an essential, high-affinity Pnr binding site in the *Dm-*DCE is conserved in the DCEs of the other species (Figure S2). Note that the three conserved Pnr binding sites are clustered in a region of the DCE that is required for activity and is sufficient in D. melanogaster to direct weak expression by itself [30]. Expression of *sc* mediated by the *Dm-*DCE is restricted dorsally through the repressor activity of Ush that associates with Pnr to prevent activation [32,45]. In gain-of-function *pnr* alleles that are insensitive to Ush, activity of the *Dv-*DCE and the *Dq-*DCE, like the *Dm-*DCE, expands dorsally. We have cloned most of the open reading frame of *pnr* from D. quadrilineata and found that, as in *D. virilis,* the two zinc fingers are perfectly conserved (Figure S3), suggesting that *Dq-*Pnr and *Dv-*Pnr may also bind Ush within their respective species [31,32,45]. Hence, it is most likely that Pnr and Ush are direct, evolutionarily conserved regulators of the DCE within Drosophilidae. Indeed the expression domain of *pnr,* as well as other upstream regulators, has been found to be conserved in other families of flies [23,27]. Even Pnr from the mosquito Anopheles gambiae is able to regulate *ac-sc* in transgenic *D. melanogaster,* suggesting conservation of *pnr* function throughout the Diptera [26].

### Morphological Diversification through *cis*-Regulatory Evolution

D. quadrilineata is phylogenetically distant from D. melanogaster and displays four instead of two DC bristles. Our results demonstrate that this secondary gain is partly due to evolution of the *cis-*regulatory sequence that drives *sc* expression at the DC site. A *Dq-*DCE-*sc* minigene, present in transgenic mutant D. melanogaster devoid of the endogenous DC proneural cluster of *ac-sc* expression, is not only able to rescue posterior bristles, but also allows development of more anterior bristles. It thus mimics the DC phenotype of D. quadrilineata itself. Expression driven by the *Dq-*DCE in D. melanogaster extends anteriorly in a domain that is longer and thinner. Although we have been unable to test the *Dq-*DCE in D. quadrilineata itself, it is active in D. melanogaster in a domain that is similar to the DC domain of *sc* expression in D. quadrilineata visualized by in situ hybridisation. This suggests that the *Dq-*DCE autonomously reproduces an expression pattern similar to the endogenous one in D. quadrilineata. Expression of *sc* mediated by the *Dm-*DCE is restricted laterally through lack of Pnr, dorsally through the repressor activity of Ush and posteriorly through the antagonistic activity of Islet [32,45,46], but it is not yet known what restricts expression in an anterior direction. The anterior expansion seen with the *Dq*-DCE indicates that this sequence may be at least partially insensitive to whatever factors limit anterior expression driven by the *Dm-*DCE. Alternatively it may contain new information not present in the other species.

Our observations demonstrate an altered response of the D. quadrilineata sequence to the upstream regulators of D. melanogaster. This response should reside in the sequence of the *Dq-*DCE itself that is sufficient to modify the phenotype of D. melanogaster when used to drive *sc*. Thus the exchange of a single, well-defined enhancer is sufficient, not only to reproduce an expression pattern, but also to partially transform a morphological trait of one species into that of another. We propose that a change in *cis,* within a pre-existing regulatory element of *sc,* contributed to the evolution of the bristle pattern observed in D. quadrilineata by altering the region where it is expressed.

The *Dv-*DCE, in *D. melanogaster,* drives expression in a larger cluster that expands predominantly in a dorsal direction. A *Dv-*DCE-*sc* minigene, however, allows the development of only two bristles positioned at the correct locations. The most likely explanation for the fact that the expanded expression driven by *Dq-*DCE-*sc* leads to additional bristles, whereas that of the *Dv-*DCE-*sc* does not, is probably linked to the different locations of the cells expressing *sc*. It seems that, in *D. melanogaster,* the region anterior to the two DC bristles is competent to produce bristles. This region is situated between the domains of expression of *sr,* a repressor of macrochaete development, and overlaps a band of expression of *wingless (wg)*, a gene encoding a secreted factor that is required to maintain *sc* expression and to repress *sr* [47,48]. It is possible to select for additional anterior DC bristles, but not for macrochaetes on either side of the DC row where *sr* is expressed but *wg* is not [34]. Notably, anterior DC bristles were present in the ancestor common to D. melanogaster and D. virilis [18,35]. The curved shape of the *Dq-*DCE–driven expression domain means that it avoids overlap with the domains of expression of *sr* and shows significant overlap with that of *wg*. Therefore only the *Dq-*DCE drives expression in an anterior location that is competent to produce bristles.

Nevertheless transgenic D. melanogaster expressing *Dq-*DCE-*sc* do not perfectly reproduce the bristle pattern of *D. quadrilineata.* The anterior-most DC bristle, the scapular bristle, is absent. This bristle is situated in the prescutum, anterior to the transverse suture. It may be that this difference is attributable to changes in factors that negatively or positively regulate the enhancer in *trans*. It is also possible that full enhancer activity requires sequences on either side of the fragment tested. Additionally, the modification of *cis*-regulatory elements lying elsewhere within the *D. quadrilineata ac-sc* complex could also have contributed to the emergence of the additional bristles. However, it is equally possible that other extraneous factors are responsible that cannot be controlled for in these experiments. For instance, it has been shown that differences in the timing of bristle precursor formation between species can influence the development of macrochaetes [24].

### Phenotypic Stability and Enhancer Evolution

The two DC bristles resulting from the activity of *Dv-*DCE-*sc* are situated at exactly the correct positions despite the fact that the *Dv-*DCE drives expression in a cluster of cells that is larger and displaced dorsally when compared with that of D. melanogaster. Thus the fly can compensate for this degree of imprecision in *sc* expression at the DC site. The explanation for this probably lies in the manner in which the bristle precursors are selected from the proneural cluster. Notch-mediated lateral signalling allows the selection of only two cells destined to become precursors with the appropriate spacing [49]. However, the choice of these cells is not random, but biased by external factors such as the repressors *emc* and *sr,* whose activity causes the precursors to arise at similar positions within the DC cluster of all individuals [34,50,51]. Their site of origin is in fact located within the region of overlap of expression driven by the *Dm-*DCE and the *Dv-*DCE. Positioning of bristle precursors thus results from restricted expression of *sc* in the proneural clusters as well as other cues that constrain the choice of precursor cell. Together, these two inputs lead to a robust patterning mechanism that is resistant to mild perturbations such as the shifting of the proneural cluster observed for *Dv-*DCE activity.

The ability of poorly conserved enhancers to drive expression of reporter genes in homologous tissues when transferred between species of similar morphology has been widely documented in the literature [52–57]. Where a detailed comparison of enhancer activity allowed a rigorous assessment of the degree of conservation, two different outcomes have been observed. On the one hand, transferring enhancers between related species of *Drosophila* (e.g., *even-skipped*), or of nematodes (e.g., *lin-48*) revealed a perfect conservation of activity [58,59], a phenomenon attributed to stabilizing selection [58]. On the other hand, the regulatory regions exchanged between species of sea urchins (e.g., *endo-16*) or ascidians (e.g., *Otx*) did not perfectly recapitulate the endogenous expression pattern [60,61]. The DCEs from D. eugracilis and D. virilis behave like the latter: they drive reporter gene expression in a cluster of cells that is not perfectly co-incident with that of the endogenous DC cluster. The slightly different expression patterns could be due to the divergent sequences, or could result from co-evolution between the enhancer and its regulatory environment [62–65]. Indeed earlier experiments have hinted that co-evolution between Pnr and its target sequences may be occurring [66].

### Role of Selection in Shaping Bristle Patterns

The role of the sensory macrochaetes in behaviour is not known. Many species of Acalyptrata have ancient stereotyped patterns in which the number and precise position of each bristle is invariant [18]. The bristle patterns of the Drosophilidae are remarkably conserved, and the majority of the nearly 4,000 species have two DC bristles [67,68]. The evolutionary stability of the many bristle patterns suggests a role for selective forces to maintain them. D. quadrilineata is unusual among Drosophilidae in having four or five DC bristles. The anterior-most DC bristles would allow additional positional sensory input, and it is possible that they confer a selective advantage. However, it is important to note that not all morphological change needs be driven by selection. Kimura proposed a neutral theory of molecular evolution in which mutations with null or negligible effect can become passively fixed in populations [69]. Similarly, natural selection alone may not explain the infinite number of subtle morphological variations displayed by the many species of *Drosophila* described [70]. Exploratory behaviour is an intrinsic property of biological systems [2], and one may therefore also speculate that evolution can proceed through a series of viable, seemingly useless, phenotypes.

## Materials and Methods

### Cloning of *sc* and *pnr* from D. quadrilineata.

For *sc,* a primer pair was designed against the regions coding for the conserved Sc N-terminal (GYQHIMP) and C-terminal (EEILDYIS) motifs (*sc*-forward 5′-CGC TAY CAG CAC ATH ATG CC-3′ and *sc*-reverse 5′-DAT ATA GTC GAG DAT YTC CTC-3′). Using genomic DNA as a template, these primers amplified a 1,010–base pair (bp) PCR product. For *pnr,* two primer pairs were designed in conserved regions to amplify small fragments of exon 2 or exon 4 (Exon2-forward 5′-GCG GCG ACT ACC ACA ACG T-3′ and Exon2-reverse 5′-GGC CGA TTC ATG CCG TTC AT-3′; and Exon4-forward 5′-GGA GGC GAG TGC CAC CAA-3′ and Exon4-reverse 5′-GAC ATT GTG CTG ATG ATG GTA-3′). Next, the D. quadrilineata sequences obtained were used to design a specific forward primer in exon 2 (5′-TAT GGA CTT TCA GTT TGG CGA-3′) and a specific reverse primer in exon 4 (5′-GTA GCA GTT ATT CAC GTA GTC-3′), amplifying a 1,033-bp *pnr* cDNA by RT-PCR.

### Cloning of the DC enhancers and transgenesis.

Specific PCR primers used to amplify the D. melanogaster and D. virilis enhancers were designed according to previously published sequences (*melano*-forward 5′-GAA GCA CTT AAC GCC AAA AGT G-3′ and *melano*-reverse 5′-GAC GAA ATG GAA ATT TGT CAA TTC-3′; and *virilis*-forward 5′-ACG GCC GGC ATT TAT TTA CTT-3′ and *virilis*-reverse 5′-GGC CAA CTT TCA GTT TTG ATC-3′). For *D. eugracilis,* the forward primer was designed in the region coding for the conserved NARQSGWW C-terminal motif of the neighbouring gene *yellow* (5′-ATG CCC GCC AAT CTG GGT GGT G-3′) and the reverse primer in a conserved region downstream of the DCE (5′-GGA AAT TTG TCA ATT CTC ACC TGG C-3′). A 2,784-bp PCR product was cloned and sequenced. A 2,073-bp ClaI-NcoI subfragment containing the DCE was then used for expression analysis. For *D. quadrilineata,* two primer pairs were designed in short regions showing a high level of conservation between *D. melanogaster, D. virilis,* and D. eugracilis to amplify and sequence small PCR products corresponding to the 5′ (upstream) or 3′ (downstream) ends of the DCE (upstream-forward 5′-GCA AAA CAA CAC TTG CTC TAT T-3′ and upstream-reverse 5′-TAA ACC GCA AAT TAG CCA CAC-3′; and downstream-forward 5′-CAT GGT TTA ATT AAA AGG TTA TTC-3′ and downstream-reverse 5′-GAA ATT TGT CAA TTC TCA CCT G-3′). These sequences were then used to design two D. quadrilineata–specific primers amplifying a 3,272-bp DCE (forward 5′-TAT CCA ACT CTT CAC TCT CCA-3′ and reverse 5′-AGT ATC AGA GTA GCC GAA AGT-3′). The DCEs were cloned in the pStinger vector [71]. Transgenes were introduced into *yw* flies as described [72].

### Immunohistochemistry and in situ hybridisation.

Third instar larval wing disc were processed according to classical protocols. For ß-Gal staining, we used a 1:200 dilution of the 40–1a mouse primary antibody (Developmental Studies Hybridoma Bank, Iowa City, Iowa, United States), and a 1:500 dilution of secondary antibody conjugated to Alexa-647 (goat α-mouse antibody; Molecular Probes, Eugene, Oregon, United States). For GFP staining, we used a 1:500 dilution of a rabbit α-GFP antibody conjugated to Alexa-488 (Molecular Probes). The actin of the discs was stained with a 1:200 dilution of phalloidin conjugated to rhodamine (Molecular Probes). In situ hybridisation reactions were performed with species-specific Dig-RNA probes detected with a 1:2,000 dilution of an α-Dig mouse monoclonal antibody conjugated to AP (Roche, Basel, Switzerland).

### Bristle rescue experiment.

The open reading frame of the pStinger GFP [71] was replaced by the open reading frame of *D. melanogaster sc*. The DCEs were subsequently cloned into the poly-linker. Homozygous *ac^mtg^* females (white eyed) were crossed to *w^−^* males bearing one autosomal copy of the *sc* minigene (marked with *w^+^*). Male progeny carrying the paternal *sc* minigene (red eyed), were scored for rescue of DC bristles. All crosses were raised at a constant temperature of 25 °C.

## Supporting Information

Figure S1 Detailed Phenotype of the *scute* Minigene Experiment The legend of the top pictures is as in Figure 2. The table shows the number of hemithoraces in each phenotypic category for four independent insertion lines of each genotype indicated.
(200 KB DOC)

Figure S2 Conservation and Variation between Distantly Related DCE Sequences (A) The legend of the diagram is as in Figure 4. The alignable regions are named R1, R2, R3, and R“*Dq-Dv*”. The corresponding alignments were performed with ClustalW [73].
(B) Sequence alignment of the DCEs from D. melanogaster and D. eugracilis. The poorly conserved central region is shown in green. In (A) and (B) the putative Pnr binding sites are highlighted in grey when they are found in one sequence only; in black when they are found in two sequences; and in red when they are conserved between all four species.
(119 KB DOC)

Figure S3 Sequence Comparison of Scute and Pnr Proteins The alignments of the *D. virilis, D. quadrilineata,* and D. melanogaster protein sequences were performed with ClustalW [73]. The bHLH domain of Scute and the two zinc fingers of Pnr are underlined. Residues known to be crucial for the interaction with Ush are highlighted in red in the D. melanogaster Pnr sequence.
(33 KB DOC)

### Accession Numbers

The GenBank (http://www.ncbi.nlm.nih.gov/) accession numbers for the D. quadrilineata sequences are DQ992393 (DCE), DQ992392 *(scute),* and DQ992395 *(pannier)*. The accession number for the D. eugracilis DCE is DQ992394.

## Abbreviations

bp: base pair
DC: dorsocentral
DCE: dorsocentral enhancer
*De*-DCE: dorsocentral enhancer of *Drosophila eugracilis*
*Dm*-DCE: dorsocentral enhancer of *Drosophila melanogaster*
*Dq-*DCE: dorsocentral enhancer of *Drosophila quadrilineata*
**Dv-*DCE*: dorsocentral enhancer of *Drosophila virilis*
DVM: dorsoventral muscle
kb: kilobase
Myr: million years
