# Supplementary material for: Two or Four Bristles: Functional Evolution of an Enhancer of scute in Drosophilidae
Source: PLoS Biol. 2006 Nov 14;4(12):e386. doi: 10.1371/journal.pbio.0040386 (PMC1635746; doi:10.1371/journal.pbio.0040386)
Supplement: Supplementary file 1 [file pbio.0040386.sg001.doc]

**Figure S1**

| *ac^mtg^* / Y ;  ***Dm***-DCE-*sc* / + | **#1** |  |  | 2 | 123 |  | 1 |  |  |  |  |  |  |
| --- | --- | --- | --- | --- | --- | --- | --- | --- | --- | --- | --- | --- | --- |
|  | **#2** |  |  | 4 | 104 | 1 | 1 |  |  |  |  |  |  |
|  | **#3** |  |  | 3 | 105 |  |  |  |  |  |  |  |  |
|  | **#4** |  |  |  | 106 |  |  |  |  |  |  |  |  |
| *ac^mtg^* / Y ;  ***Dv***-DCE-*sc* / + | **#1** |  |  |  | 118 |  |  |  |  |  |  |  |  |
|  | **#2** |  |  |  | 116 |  |  |  |  |  |  |  |  |
|  | **#3** |  |  |  | 104 |  |  |  |  |  |  |  |  |
|  | **#4** |  |  |  | 101 |  | 1 |  |  |  |  |  |  |
| *ac^mtg^* / Y ;  ***Dq***-DCE-*sc* / + | **#1** |  |  |  | 100 | 5 | 6 | 4 |  |  | 1 |  |  |
|  | **#2** |  |  |  | 84 | 13 | 16 | 6 | 7 |  |  | 1 |  |
|  | **#3** |  |  |  | 46 | 28 | 24 | 5 | 6 |  | 10 | 9 |  |
|  | **#4** |  |  |  |  | 4 | 11 | 2 | 12 | 8 | 17 | 38 | 25 |
