# Supplementary material for: Two or Four Bristles: Functional Evolution of an Enhancer of scute in Drosophilidae
Source: PLoS Biol. 2006 Nov 14;4(12):e386. doi: 10.1371/journal.pbio.0040386 (PMC1635746; doi:10.1371/journal.pbio.0040386)
Supplement: Supplementary file 2 [file pbio.0040386.sg002.doc]

**Figure S2**

(**A**) Sequence comparison of DCEs from *D. melanogaster*, *D. virilis*, and *D. quadrilineata*:

Region 1 :

*melanogaster*-------TTAACGAGAAACA------AGAATATC---------------------------------

*virilis*------------TTAACGAGAAACGTA-CGCGGAATTGGACAGCCCCAGCCCCAGCCCCAGCCACAGTCATC

*quadrilineata*------TTAACGAGAAACGCATCGCAGG------CAGCCGGAGTCACTGTTGTTGCCACTGCCACT

-------------------************--------*--------------------------------------

*melanogaster*--------C-------------------------------------------------TACGTGTGG

*virilis*-------------CCAGCGACAGCGACAGAGTCCCTGCCCCTGTCCGAGCTAAGCACAACGCATACGTGTGG

*quadrilineata*------GCCACTGACACTGACACTGCCACTGCCTGTT----------GCATGTTGCATACGTGTGG

--------------------*-------------------------------------------------*********

*melanogaster*-------CTAATTTGCGGTT-A-----------------------AGG--------------AAG--

*virilis*------------CTAATTTGCGGTT-GCCCGAATGATGATTTACAT----AGG--------------CAGCC

*quadrilineata*------CTAATTTGCGGTTTGCTTGTTGCATGATTTACATACATAGGTCAAACCTTTGCTGCGGCT

-------------------*************-------------------------***----------------*--

*melanogaster*--------GCC---CC----A------------TGATAT----------------ATAAAG------

*virilis*------------GGCCAGATC----AGCACAAACCGAGTGAGATTCAGCA----------GCAGAC------

*quadrilineata*------AACCAGCCCCCAAACTGCCAACAGATCAACACCAAAAGTGCCAAAAGCGCAAAAAATTAA

---------------------**----*----*--------------*-*-------------------*-*-------

*melanogaster*-------CCAAAAAATAAAGCA--------------AAA----------------------------

*virilis*------------CCAAAAAAAAAAGAAGAAGAAGAAGAAGCAAA----------------------------

*quadrilineata*------CAAAAAAAAAAAATATAAGAAAGAAAAACAAATATATGGAAAAAATGTTGGCAGAAATTG

-------------------*-******-***--*--------------***----------------------------

*melanogaster*---------------CCGCA----------GCAGTTCATTCAAATTTT-TTGTGGC-----CA----

*virilis*--------------------CCGCAC-------ATGCAAATTATAGAAATTTCATCGTGGCGCTGGCAGAGC

*quadrilineata*------GTAAGCGACCGCAGTTTGGAAATGCAAATTATAGAAATTTTGCACTGGC--TGACGGC-C

---------------------------*****----------***--*-**--******-----****-----*-----

*melanogaster*---------AAACAAATATTAAGATA--------------------------AAAGTCAG-----T-

*virilis*------------TTAAACAAATATTGAGATA--------------------------GTCGTCCGATAACT-

*quadrilineata*------TAAAACAAATATTGCGTTTGTAGCGCCATCTCATTCGCACCCACAAGCGCCATCTCGCTC

---------------------***********-----------------------------*-*---*-*-------*-

*melanogaster*---------------GG-----GGACTATAATTTG----AGTT---------ATTCAAATGCAATAAATC

*virilis*--------------------GGCC--AGGACTATCATTAGTT-CAGCTGCAACGCATATTCAAATGCAATAAATC

*quadrilineata*------TCGCCTTGAGTCTAAGCACTATCATTAGTGACGGTTTT--CACTTATTCAAATGCCATAAATC

----------------------------*-----*-*****-***-*-----*-*---------**********-*******

Region 2 :

*melanogaster*-------TTGCTAATTTTACGTTAGACGCCCTTTAATTTCACGCTGC------CATTTGTGGCTTCCG

*virilis*------------TTGCTAATTTAATGCTAGACGGCGTT-AATATCAAGCTGC------CATTTATGGCTTCCG

*quadrilineata*------TTGCTAATTTACTGCTAGACGGCGTT-AATAACAAGCTGCCATTGCCATTTATGGCTTCCG

-------------------**********---*-******-*-**-***--**-*****------*****-*********

Region 3 :

*melanogaster*-------GATATGACAAA-----CACGCGTGAATAG--------------CGTCAAACGTTTTTAGA

*virilis*------------GATATGACAAG----ACACGCGTGAAGAGCGATGAGAGTGGAGCGTCAAACGATTTTGTT

*quadrilineata*------GATATGAGAAAAGAAACACGCGTGAAGAGAGTTGCGAG-----CGTCAAACGATTTTGTT

-------------------*******-**------**********-**--------------*********-****---

*melanogaster*-------GCTCTTATCGTCA-----------------TGGTTTAATTAAAAGGTTATTCTTGTAGGC

*virilis*------------ATCGTTGTCGCCAT------AACCGTTGCATGGTTTAATTAAAAGGTTATTCGTGTATGT

*quadrilineata*------ATCATTGTCGCCACAAACTGAACTGTTGCATGGTTTAATTAAAAGGTTATTCTTGTATCC

-----------------------**-***-**-----------------**********************-****---

*melanogaster*------------------------------AGGATAACATTGCGGCCGAAAGG----------ATGC

*virilis*------------CGCCAGGCGGCTGGTCTCTAACCCGGATAACCATGTAGATA---ATGCCCATAAGCAGGC

*quadrilineata*-------------------------TGCAAGGATAACCCAAGGGTTATA--AGATAACCATGCCAC

-------------------------------------------*******-----------------

*melanogaster*--------AATTGAATT-GTC-------------------------------------------TGT

*virilis*------------CGACCGAGTGAGCCGGAGAGACAGAGACAGAGACAAACACAGACAGACAGACAGACACAC

*quadrilineata*------ACATTTTGATTGAGTT-GTTGCAACAGCAACAGCAGCAGCAG---------CAGCAACAC

---------------------------------------------------------------------

*melanogaster*-------ATTG------GGGTAATTA------------TTCAGT--------GC--------CTTATCTTAAAGACTTT

*virilis*------------ACTTTTGATTGAGTTGTTTAAATCGTTGTTGCTCTCTCACGGGCTGCGATTGTTGCTTATCTTAAAGACTTT

*quadrilineata*------ACTC------GACTCATTT------------TCCTCT--------GTG-------TTTATCTTAAAGACTTT

-------------------*-*-------*--*--**---------------*--*--------*----------****************

Region *Dq-Dv* :

*virilis*------------AAATACAGCT-----ACTTAATTGAAGGCGCCAACAAGCAGCGGGCGGTTTGTGGCGTTT

*quadrilineata*------AAATAGAGCTCTACAACTTAATTGAAGGCGCCAACAAACAGCGGGCGCC------CATTT

-------------------*****-****-----**********************-*********--------*-***

*virilis*------------TGGGGCGTGGCACTTGAGTGCAATTGCTGACCTGCTGTGC--AGCT-GTTGCCAT-GTTG

*quadrilineata*------TGGGGCGTGGC----GGGTG--ACTG--GACATGCTGTGCCAAGTTCGTTGCTTCAGTCA

-------------------***********----*-***--*-**--***-********--**-*-*****----**--

*virilis*------------CCAACAAGACGCACCGTCAGATGAGAGGAGACCAAAGCAAGTTCGCTGCTTAAGTCATTC

*quadrilineata*------TTATCAGCAAATGCTG------GCGATGGGGC---AGCAAGTTCGTTGCTTAAGTC-TTT

---------------------*-**--*----*-*------*-**-*-*-*---**********-**********-**-

*virilis*------------CGTTTGTTAAGTTCGTTGCTTAAGTCATTCGTTTCACCTGATCCCCAGAGCTGCTGAGCT

*quadrilineata*------CGTTT-----------TGCCT--GTCA---GTTTTGGTTGGACGCTCGCTCT-TTGCG-T

-------------------*****-----------***-*--****---****----**--*-*--*--**--**-*-*

*virilis*------------CGCTGTTTTCAATTAAAAGCGACGCTAATTTTATCGAACAATGCAACAAATTTTATGCGC

*quadrilineata*------CGTTTTTTTCAATTAAAA---------------------------ACAATTTTTATGCGT

-------------------**-*-*************---------------------------****-*********-

*virilis*------------ATTT-AACAAACTCGTAATTGATTG--TACAA-----------AAGTGAAGG

*quadrilineata*------ATTTTAACTAAGTTGTAATTGATTTGCCACACCTTCTGGCAGCAAGTGAAGG

-------------------****-***-**-*-**********----***------------*********

(**B**) Sequence comparison of the DCEs from *D. melanogaster* and *D. eugracilis*:

*melanogaster*------TTAACGAGAAAC------------------AAGAATATCCTACGTGTGGCTAATTTGCGG

*eugracilis*--------TTAACGAGAAACCAGAAACGAGAAACGCGCGAGAATATCGTACGTGTGGCTAATTTGCGG

------------------************-------------------********-********************

*melanogaster*------TTAAGGAAGGCCCCATGATATATAAAGCCAAAAAATAAAG------CAAAAC--------

*eugracilis*--------TTAAGA----CCGCATGATTTACATAGCCAAAAAAATAAGAAAGCCCAAAACTCAGAGCA

------------------*****-----**-******-**-*-**********--***------******--------

*melanogaster*-------CGCAGCAGTTCATTCAAATT-------TTTTG---------------TGGCCAAAACAA

*eugracilis*--------GCAGAGCAGGCCATGCAAATTATAGAAATTTTGCGACCCTTCGGCTTTTGGCCAAAACAA

-------------------*--*****--***-******-------*****---------------************

*melanogaster*------ATATTAAGATAAAAGTCAGTGGGGAC-TATAATTTGAGTTATTCAAATGCAATAAATCCG

*eugracilis*--------ATATTGAGATAAAAGTCGGTCTGGCCATATAATTTGAGTTATTCAAATGCAATAAATCCG

------------------*****-***********-**--**-*-*********************************

*melanogaster*------CGGAACCAAACGAGTTGCTAATTTTACGTTAGACGCCCTTTAATTTCACGCTGCCATTTG

*eugracilis*--------GCGAACGAAACGAGCTGCTAATTCCACGTTAGACGCCCTCTAATTTCACGCTGCCATTTG

--------------------****-*******-********--**************-********************

*melanogaster*------TGGCTTCCGTTTCGTATAAGTTTGGGTCGA-TGATTTTTA**GAATTTGC----ATTTTCAA**

*eugracilis*--------TGGCTTCCGTTTCGTATGAGTTTTGCCCTTCTCATTTGAA**TATCCTGCTGCGGTTTTAAA**

------------------*****************-*****-*--*---*-****--***-*---***-----****-****

***melanogaster*------TTTGAATAT-AAAAAACCGCCGCTGCAGGTTTG-CAGC-------TTTTAAAGTGATC--**

***eugracilis*--------TTTAAGTTTTGGGAAACCATC-TTGCAGCTTCATCAGCAAACTGATTTGAGCTTAATTGA**

**------------------***-*-*-*----*****--*--*****-**---****-------***-*---*-**---**

***melanogaster*---------CGCAATCTCATTCCTAGTCAGTCGAAAAAAGGTAAGATAGAGAAAAGAACAGAAATG**

***eugracilis*--------AGGCGCCAGCTCTTTCCTAGT---TCAAGCTGAGCCACCGCTGTGCCA--CAGAGAAGTG**

**---------------------***-*-***-********---**-*----**--*-----*-*--*---*-****-****

***melanogaster*------ACTACAAAATTAAGTACCGAATTTTGTGCCATCGTAAT----------AGTTTTTCGCCA**

***eugracilis*--------CCTACAAAGTC--GTCTCAAGCTGTTTTCCAGCGGAGTTCGTCGCCTCAGTCTTTCGCTT**

**-------------------*******-*---**--*-*--*-*-*-***-**-*-*----------***-******--**

***melanogaster*------TTTCCCTT--------------TCACGGG------------------TTTAACTG-----**

***eugracilis*--------GGCCCCTCAACGGAAAAGTGGATTACGGACAGCTTGTAAACAGGGTATTTAACAGACCGC**

**---------------------****---------------*-****-------------------******-*-----**

***melanogaster*-------TTAATTATGT-----AATTAAATGTAAAGTCGTGG--AACTCGGCGTTTT----TTCTT**

***eugracilis*--------TTTCGGTATCTCAGCGAATTCAAT-TAAAGGCATGGCAAATCAAGAGTTTTATGCTCTTT**

**-------------------**---***-*-----****-***-*****-*-***--**----*-*****----*--****

***melanogaster*------GATCAAT--------------------------------------ATAC-----------**

***eugracilis*--------AATCAAAGCCTTAATTGATTGCCGAAGGTCGCAGGAAGGCGTGAAATGCGAGCTCAGCCA**

**-------------------*****---------------------------------------**-*-----------**

***melanogaster*-------------ACATA--------------------------TTTC---------CGGCGC---**

***eugracilis*--------GGGGGCCACATAAAAGCGGATCGAAACTGCAACTAGGCTTGCAATAAAAAGCGGCGCCAC**

**-------------------------*****--------------------------**-*---------******---**

***melanogaster*------------------------AAATGC----------------TATTTTTATGA-GCATTTCA**

***eugracilis*--------AAAGCCAAGCAAGTTATTAAATACACTCCGATTATTTGGTTATTTTTCCGATATATTTCC**

**------------------------------------****-*----------------*******--**---*****-**

***melanogaster*------TGT---AATTA--------TT---GAGACTAATAGGAAGAC**AGATCTTGTTTGGGTGCAG

***eugracilis*--------AGCTCAAATTAAGGCATAGTTTGGGAGAACGACAGAGGGAG**AGATCTCGATTGGGAGCAG

**-------------------*----*****--------**---****---*-**---**-********-*-*****-****

*melanogaster*------GGAAAGTTCAACTTAATCGCTCAATTTGAGATCGCCTGGTCGCTTGAGATTCGA------

*eugracilis*--------GGAAAGTGCAAGTTAATCGCTCAATTCGCGGCCTGCTGGCCCCTTGGGATGCGATCCGCT

------------------*******-***-**************-*-*--*--****-*-****-***-***------

*melanogaster*--------------CTGTAATTGAAATTTTTGCTTT--TGATCGGAGCCA--GACTTCAGACGGGG

*eugracilis*--------GGATTCGCCTGCAATTGAAATTTC-GTTTTAATGGCCGCAGTTTATGACATCGGTTGGGG

--------------------------***-***********--*-***--**--**-**-----***-**-*--****

*melanogaster*------C-------AAA---------CAAAAA-GACTTTGTTGGTGGTAGGGT------AGGATCG

*eugracilis*--------CCACTGCGAAATTGCCGGACCAGAAATGACTTTGTTGGCGGTAAGATCGTAAAAGTAAGA

------------------*-------***---------**-***-***********-****-*-*------**-*---

*melanogaster*------TAAAAAGAACTCAGAACAGAACCAGAAT-------AAAAATTCGGATAAGGATTGTG---

*eugracilis*--------GAAGAAGAACCTAGCCCAGAACCCGAAACCCAACGGAAAAGTGCGATAAAGATAGCGCGA

-------------------**-******--**--*******-***---------****-*--*****-***-*-*---

*melanogaster*------------AGCCTGTAGTCGTACAAAAAA----------AGATA--ATTCTGTCTGCAGGTA

*eugracilis*--------GCATGTAGCGTGTAGCCGCACAAAAAACGAAACGAAAAGATACAATTGTATCTGCAGGTA

------------------------***-*****-**-********----------*****--***-*-**********

*melanogaster*------CAAAA-------AAAAGGCAGATATAGTTGGC---------AGCTGTATAAAACTAATGC

*eugracilis*--------AGAAAGTAAAGCAAAAGGAAGGTAGGCTCGGCCCCGTGTGCAGCTGGATAAAACTAATGC

--------------------***-------******-**-**---*-***---------*****-*************

*melanogaster*------ACTTGC---AGTCCAATCTAATCGTTTGCCAAATGTCAAACGAGTTTCATTCATCCGGCG

*eugracilis*--------ACTTGCTGCGGTCCAATCTAATCGTTTGCCAAATGTCAAACGAGTTTCATTCATGAGGCG

------------------******----********************************************--****

*melanogaster*------ATACCCCTTCCTTTAAATCTTCCTTTTCCCTTTTTTTAAAGTAAACTGTGAGTCAGCTCA

*eugracilis*--------ATACCCCTTCC------CCTTGCCTTTCC---TTCCTAAGAT---CCGAGAG--AGTTCA

------------------***********-------***-*-*****---**--***--*---*-*-***--**-***

*melanogaster*------TTCATGGAGAACCGTGACGATAAGTCCG---CCTCTTTATTTTCGATATGACAAACACGC

*eugracilis*--------TTCATGGAGGACCGTGGCGATAAGTCCGGTTCTTCTTTATTTCCGATATGACAAACACGC

------------------*********-******-***********---*-*********-*****************

*melanogaster*------GTGAATAGCGTCAAACGTTT--TTAGAGCTCTTATCGTCATGGTTTAATTAAAAGGTTAT

*eugracilis*--------GTGAAGAGAGTCAAACGGTCGGTCGGCCTGCTTATCGTCATGGCTTAATTAAAAGGTTAT

------------------*****-**-********-*---*--*----*************-****************

*melanogaster*------TCTTGTAGGCAGGATAACATTGCGGCCGAAAGGATGCAATTGAATTGT----------CT

*eugracilis*--------TCTTGTAGGCAGGATAACACTTTGGCCGCAAGGATGCGATTGAGCTGTTGTCCCTTGGCT

------------------*******************-*--*****-********-*****--***----------**

*melanogaster*------GTATTGGGG-TAATTATTCAGTGCCTTATCTTAAAGACTTT

*eugracilis*--------GCGCTGGGGGTATTTACTCGGCCCCTTATCTTAAAGACTTT

------------------*---*****-**-***-**-*--******************
