# Supplementary material for: Two or Four Bristles: Functional Evolution of an Enhancer of scute in Drosophilidae
Source: PLoS Biol. 2006 Nov 14;4(12):e386. doi: 10.1371/journal.pbio.0040386 (PMC1635746; doi:10.1371/journal.pbio.0040386)
Supplement: Supplementary file 3 [file pbio.0040386.sg003.doc]

**Figure S3**

Scute alignment:

*virilis*------------AP---ELKTSSSSSSGGASLIKTRKYTPRGMALANASSGSSSSSSSSSCCSTSSPYNVDQ

*quadrilineata*------APTPQQPQQQHQHQLQQKSLMKTRKYTPRGMALATAAGAATCHTTATS-----APYSVDQ

*melanogaster*-------APSPLIPGGN-QNQPAGTMPIKTRKYTPRGMALTRCSESVSSLSPGSS----PAPYNVDQ

-------------------**-.-------.-.------:************:-.:-.-:.-:..:*-----:**.***

*virilis*------------SQSVQRRNARERNRVKQVNNSFARLRQHIPQSIIADLTKGGGRGPHKKISKVDTLRIAVE

*quadrilineata*------SQSVQRRNARERNRVKQVNNSFARLRQHIPQTIIADLTKGGGRGPHKKISKVDTLRIAVE

*melanogaster*-------SQSVQRRNARERNRVKQVNNSFARLRQHIPQSIITDLTKGGGRGPHKKISKVDTLRIAVE

-------------------*******************************:**:*************************

*virilis*------------YIRRLQDLVDDLNGGP---SSNSTGSCNMAQLNLCLDEETC----------------SNS

*quadrilineata*------YIRRLQDLVDDLNGGSNTSNANATAAAHVAQLNLCLDETSSQSCESSSSSTNGSSSHSHS

*melanogaster*-------YIRRLQDLVDDLNGG-----SNIGANNAVTQLQLCLDESSS----------------HSS

-------------------***************-----:*--.---::**:*****-:.------------------*

*virilis*------------SSISSSSSSLGSQQL--NHNLYYSSSLLTAAATPTALQQQQQLQRQQFPHQPLTPITLNGNT

*quadrilineata*------SSSNLSASLA--QQLYYNSNINTTALQQQQQQQQQQLQQQQQLQRQQYPNHPLMPIIPN---

*melanogaster*-------SSSTCSSS---------GHNTYYQN-----TISVSPLQQQQQLQRQQFNHQPLTALSLN---

-------------------**-.-*:*---------.-*----------------***********:-::**-.:--*---

*virilis*------------LPTAATTTTAATAAAATTTTTTTGGCHSPTSSFNSSMSFDSGTYEAQPQQLSPPVAPM--

*quadrilineata*-----------TNMPLVATTAVVAPGGTTTVCHSPTSSFNSSMSFDSGTFEAAPQQLSPTEAIISS

*melanogaster*--------TNLVGTSVPGGDAGCVSTSKNQQTCHSPTSSFNSSMSFDSGTYEGVPQQISTHLDRLDH

-------------------------.------:.-.:.--.---******************:*.-***:*.----:.-

*virilis*----------------------DAQLQSKFEPYEHFQLDEEDCTPDD

*quadrilineata*------GTSPASSSTLDAQLQLKFEPYEHFQLDEEDCTPDE

*melanogaster*----------LDNELHTHSQLQLKFEPYEHFQLDEEDCTPDD

------------------------------:***-******************:

Pannier alignment :

*virilis*------------QLQVAAVGASSKMYHSSSAAAAAAYTDLAAAGGAAS------VSGYHHQQAVNAPVYVPSN

*quadrilineata*------QLQVAAVAASSKMYHSSSAAAAAAYTDLAA-GSAAS------VSGYHHQQAVNAPVYVPSN

*melanogaster*-------ASASATHVAAVKMYHSS---AVAAYTDLAAAGSAASAGVGVGVSGY-HQQAVNAPVYVPSN

-----------------------*:--*:-******---*.********.*.***..----****-**************

*virilis*------------RQYNHVAAHFGS-AAAAQNAWTTDSFGSAHAQLPAQFYTQNAAVMMAPWRSAYDPTGFQR

*quadrilineata*------RQYNHVAAHFGSAAAAAQNAWTTDSFG--TAQLPAQFYTQN-AVMMAPWRSAYDPTGFQR

*melanogaster*-------RQYNHVAAHFGS--AAAQNAWTTEGFG----SAHAQFYSPNAAVMMGSWRSAYDPSGFQR

-------------------************--*********:.**----.--****:-*-****..*******:****

*virilis*------------SSPYESAMDFQFGEGRECVNCGAISTPLWRRDGTGHYLCNACGLYHKMNGMNRPLIKPSK

*quadrilineata*------SSPYESAMDFQFGEGRECVNCGAISTPLWRRDGTGHYLCNACGLYHKMNGMNRPLIKPSK

*melanogaster*-------SSPYESAMDFQFGEGRECVNCGAISTPLWRRDGTGHYLCNACGLYHKMNGMNRPLIKPSK

-------------------************************************************************

*virilis*------------RLVSATATRRLGLCCTNCGTRTTTLWRRNNDGEPVCNACGLYFKLHGVNRPLAMRKDGIQ

*quadrilineata*------RLVSATATRRMGLCCTNCGTRTTTLWRRNNDGEPVCNACGLYFKLHGVNRPLAMRKDGIQ

*melanogaster*-------RLVSATATRRMGLCCTNCGTRTTTLWRRNNDGEPVCNACGLYYKLHGVNRPLAMRKDGIQ

-------------------**********:*******************************:*****************

*virilis*------------TRKRKPKKTGSGSTAGTSSGS--GSTTEAIKECKEEHDLKPTLSLERHSLNKLHTDLKSS

*quadrilineata*------TRKRKPKKTGSGSSSGAGVGS--GSSADPIKDCKEEHDLKPTLSLERQSLSKLHTDLKST

*melanogaster*-------TRKRKPKKTGSGSAVGAGTGSGTGSTLEAIKECKEEHDLKPSLSLERHSLSKLHTDMKSG

-------------------*************:-*:.-**--**:-:.**:*********:*****:**.*****:**-

*virilis*------------LAG----RAVSSLMGQHHSQQPQQQS------APSHQQCFPHYAQQHQQQQQQQQQQ---

*quadrilineata*------LGN----SAASSLMSHHHAQQQQQQPQQQQQPPPSHQQCFPLYAQQQQQQQQQHQQQQQQ

*melanogaster*-------TSSSSTLMGHHSAQQQQQQQQQQQQQQQQQQQQSAHQQCFPLYGQTTTQQQHQQHG----

--------------------..-----.--*---:::-**-***--------.:******-*.*---***:*::-----

*virilis*----------------SQHQHLSHQSQLSARQLHGAG-SQLYTPSSSSA---ASAYTTHSSNAADTPTLSNG

*quadrilineata*------QQHQSQHPQHSHQSQLSARHLHGTG-SQLYTPSSSSA---ASAYTTHSSNAPDTPTLSNG

*melanogaster*-----------HSMTSSSGQAHLSARHLHGAAGTQLYTPGSSSGGGSASAYTSHSA---ETPALSNG

------------------------.----*-*::****:***:.-:*****.***...:*****:**.:**:*******

*virilis*------------TPSPHYQHHHLHAAAAASSS--HGHHAAAAHHHIHAAAAAAAYGVKTEASATNYDYVNNC

*quadrilineata*------TPSPHYQHHHLHAAATSGSIGHHAAAAAASHHHIHAAAAAAAYGVKTEASATNYDYVNNC

*melanogaster*-------TPSPHYQHHHHLGGTHG-----HHVTAAAAHHHFHAAAAVAAYGVKTEASATNYDYVNNC

-------------------**********--..:-.-----*---***:***:*****.********************

*virilis*------------YFGASFGALGG-AASSALAGGATSDLAG

*quadrilineata*------YFGASFGALGG-AATSAMAGGASSELAG

*melanogaster*-------YFGGTFGALGGAATTTAMAGGAASELAG

*-------------------****.:******.*:::*:****:*:***
